# Socio-demographic characteristics associated with perceived social support among parents of children aged 0–7 years: the CIKEO study

**DOI:** 10.1186/s12889-022-14830-1

**Published:** 2022-12-27

**Authors:** Irene N. Fierloos, Dafna A. Windhorst, Yuan Fang, Harrie Jonkman, Matty R. Crone, Clemens M. H. Hosman, Siok Swan Tan, Hein Raat

**Affiliations:** 1 Department of Public Health, Erasmus University Medical Center, Doctor Molewaterplein 40, 3015 GD Rotterdam, The Netherlands; 2 Department of Cognitive Neuroscience, Donders Institute for Brain, Cognition and Behaviour, Radboud University Medical Center, Kapittelweg 29, 6525 EN Nijmegen, The Netherlands; 3 TNO Child Health, Sylviusweg 71, 2333 BE Leiden, the Netherlands; 4 Verwey-Jonker Institute, Kromme Nieuwegracht 6, 3512 HG Utrecht, The Netherlands; 5 Department of Public Health and Primary Care, Leiden University Medical Center, Albinusdreef 2, 2333 ZA Leiden, The Netherlands; 6 Department of Health Promotion, Maastricht University, Peter Debyeplein 1, 6229 HA Maastricht, The Netherlands; 7 Department of Clinical Psychology, Radboud University, Thomas van Aquinostraat 4, 6525 GD Nijmegen, The Netherlands; 8 Hosman Prevention and Innovation Consultancy, Knapheidepad 6, 6562 DW Berg en Dal, The Netherlands

**Keywords:** Social support, Parenting, Empowerment, Population characteristics, Socioeconomic differences

## Abstract

**Background:**

Social support has been associated with numerous positive outcomes for families’ health, wellbeing and empowerment. This study examined which socio-demographic characteristics are associated with perceived social support among parents of children aged 0–7 years.

**Method:**

Cross-sectional data of 1007 parents of children aged 0–7 years, gathered in the CIKEO cohort study in the Netherlands, were analysed. Social support was assessed with the Multi-dimensional Scale of Perceived Social Support (MSPSS). Linear regression models were used to examine associations between socio-demographic characteristics and perceived social support.

**Results:**

The mean age of the participants was 34.1 years (SD = 5.1); 92.9% were mothers. The multivariable regression model showed that fathers (β: -0.15, 95% CI: − 0.22, − 0.08), parents with a low educational level (β: -0.12, 95% CI: 0.18, − 0.06), parents with a low income (β: -0.10, 95% CI: − 0.19, − 0.01), unemployed parents (β: -0.14, 95% CI: − 0.20, − 0.07), and parents of older children (β: -0.07; 95% CI: − 0.13, 0.00) perceived lower levels of social support. Interaction analyses showed that parents with a migration background and a low educational level were particularly susceptible to perceiving lower levels of support (β: -0.34, 95% CI: − 0.52, − 0.15).

**Conclusion:**

Fathers, parents with a low educational level, parents with a low income, unemployed parents, parents of older children, and parents with both a migration background and a low educational level are at increased risk of perceiving lower levels of social support.

**Implications:**

We recommend to develop, implement and evaluate intervention strategies to strengthen perceived social support among the abovementioned subgroups of parents, in order to improve families’ health, wellbeing and empowerment.

**Trial registration:**

NTR7607 in the Netherlands trial registry.

**Supplementary Information:**

The online version contains supplementary material available at 10.1186/s12889-022-14830-1.

## Introduction

Social support has been associated with numerous positive outcomes for parental health and wellbeing, including better coping mechanisms, reduced stress, a decreased risk of depression, a decreased risk of cardiovascular diseases, and lower mortality rates [1–7]. Furthermore, social support has been associated with positive outcomes for parenting, including a higher parenting sense of competence and more positive parenting behaviour [8–17]. Recently, there has been increased attention for policies that empower parents to solve parenting issues within their social networks and communities [18–20].

Social networks may help to prevent, solve and alleviate parenting issues by providing access to various types of social support, which can be distinguished as instrumental, informational, appraisal and emotional support [21–23]. Instrumental support relates to financial, material and in-kind support to assist with tangible needs, such as assistance with child care. Informational support relates to the availability of advice and information. Appraisal support relates to the provision of feedback and support with decision-making. Emotional support relates to the availability of love, sympathy, esteem, trust, listening and understanding [21–24]. A distinction can be made between ‘received’ and ‘perceived’ social support [25]. ‘Received’ social support can be defined as the actual amount of social support one receives, and ‘perceived’ social support can be defined as the extent to which an individual believes social support is available when needed [5, 25]. Previous research suggests that perceived social support is more strongly related to health and wellbeing than received social support [5, 25]. 

Given the beneficial outcomes of social support for families’ health, wellbeing and empowerment, it is important to know which parents may be at risk of perceiving a lack of social support. Previous studies suggest that several groups of parents, including single parents, parents with a low socio-economic position, and parents with a migration background, may perceive lower levels of social support [26–31]. However, this is based on a relatively small number of studies, using different measures to assess perceived social support, with varying levels of validation [26–31]. An important shortcoming is that social support measures used in previous studies mainly addressed the instrumental dimension of social support, but paid little attention to the emotional and appraisal dimensions of support [26, 27, 29–31]. In addition, previous studies often did not distinguish between different sources of social support, such as support provided by family, a special person and friends [26, 27, 29–31]. There is a need to gain a better understanding of the distribution of perceived social support among parents, which relates to all dimensions of support and to different sources of support.

 This study will examine which socio-demographic characteristics are associated with perceived social support among parents of children aged 0–7 years. We use a validated measure which includes the emotional and appraisal dimension of social support and distinguishes between support provided by family, a special person and friends [32]. Achieving a solid overview of the socio-demographic characteristics of parents who perceive lower levels of social support may enhance the identification of parents in need of intervention strategies to increase social support and related health, well-being and empowerment [33].

## Methods

### Design

This cross-sectional study used baseline data of an observational cohort study embedded in the Consortium Integration Knowledge promotion Effectiveness Of parenting interventions (CIKEO) [34]. The CIKEO study was originally designed as a naturalistic effect evaluation, to investigate associations between exposure to elements of various types of parenting support and parent and child outcomes [34].

### Ethical considerations

The Medical Ethics Committee of the Erasmus Medical Center, Rotterdam, decided that the rules laid down in the Dutch Medical Research Involving Human Subjects Act (in Dutch: ‘Wet Medisch-wetenschappelijk Onderzoek met mensen’) did not apply to the research proposal (proposal number MEC-2017- 432), that there were no objections to the execution of this study, and approved that the results of the study could be submitted to scientific journals (Letter NL/sl/321518; 24/07/2017). The CIKEO cohort study was registered as NTR7607 in the Netherlands Trial Registry [34].

### Sample/ participants

Participants were enrolled between October 2017 and December 2019 by two recruitment methods. First, two preventive community Youth Health Care (YHC) organizations in the area of Rotterdam and Dordrecht sent invitation letters to 6506 parents/caregivers of children aged 15 months to 6 years in their registries. In the Netherlands, all children are registered at their local community youth health care organization, regardless of whether or not they use youth health care services. In addition, parents of children aged 0–7 years who were planning to participate in a preventive parenting program were recruited via advertisements and providers of preventive parenting programs [34]. All participants received an information letter, an informed consent form and a questionnaire. The parent/caregiver who was spending most time with the child was invited to complete the questionnaire. Participation was voluntary. Parents/caregivers could return the informed consent form and the questionnaire in a pre-paid envelope or via the internet. All parents/caregivers who provided written informed consent and a completed questionnaire were enrolled in the study.

**Fig. 1:**
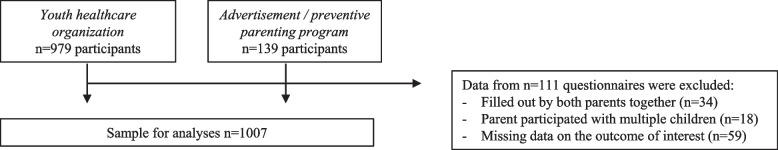
Flowchart of the inclusion process of the CIKEO cohort study and the population for analysis

 In total, 1118 parents participated in the baseline measurement (Fig. 1). The response rate for the 979 participants recruited via YHC organizations was 15%. The response rate for the other 139 participants was unknown due to recruitment via advertisements. Data from 34 questionnaires completed by two parents together were excluded, as this study aimed to examine their individual perception of social support. In total, 18 parents participated twice in the study with multiple children, data from their second questionnaires were excluded. Participants with missing information on the outcome of interest (*n* = 59) were also excluded. Hence, the population for analysis consisted of 1007 participants.

### Data collection

#### Social support

Perceived social support was measured by the widely used 12-item Multi-dimensional Scale of Perceived Social Support (MSPSS) [32]; a validated Dutch translation was available [35]. Previous validation studies showed satisfactory internal reliability among diverse populations, with Cronbach’s alpha coefficients ranging between .74 and .95 [32, 36–39]. The MSPSS consists of three subscales assessing perceived social support provided by family members, a special person, and friends, by items such as: ‘I get the emotional help and support I need from my family’; ‘My friends really try to help me’; ‘There is a special person in my life who cares about my feelings’. Each subscale consists of four items. The scale uses a 7-point Likert scale, ranging from 1= ‘very strongly disagree’ to 7= ‘very strongly agree’. The twelve items add up to a total score, which is then divided by twelve. Scores for the subscales are calculated by adding up the four items and dividing by four. A score of 1 indicates the lowest level of perceived support, and a score of 7 indicates the highest level of perceived support [32]. There are no established population norms on the MSPSS, but scale response descriptors can be used as a guide [40]. Scores ranging from 1.0 to 2.9 can be considered ‘low’ support, scores ranging from 3.0 to 5.0 can be considered ‘moderate’ support, and scores ranging from 5.1 to 7.0 can be considered ‘high’ support [40]. For the logistic regression analysis, ‘low’ and ‘moderate’ MSPSS scores (< 5.1) were categorised as ‘low to moderate’ perceived social support.

### Socio-demographic characteristics

The following socio-demographic characteristics were studied: age of the responding parent (in years), gender of the responding parent (female/male), educational level of the responding parent, household income, employment status of the responding parent, migration background of the responding parent, family situation (living with/without a partner), number of children in the household (one/two/more than two), age of the child (in years), and gender of the child (girl/boy).

 The educational level of the responding parent was assessed by his/her highest completed education and was reclassified into three categories based on the International Standard Classification of Education 2011 [41]. Level 6–8 (bachelor, master, doctoral or equivalent) was categorised as ‘high’; level 3–5 (upper secondary education, post-secondary non-tertiary education, short-cycle tertiary education) was categorised as ‘middle’; level 0–2 (no education, primary education, lower secondary education) was categorised as ‘low’ [41]. Net monthly household income was categorised as low (<€2000), middle (€2000–€3200), or high (>€3200) [42]. Parents were asked to specify their current employment status as: ‘working fulltime’, ‘working part-time’, ‘stay-at-home parent’, ‘unemployed’, ‘incapacitated’, ‘studying’, and ‘other’. ‘Unemployed’ and ‘incapacitated’ were categorized as ‘unemployed’ and ‘studying’ and ‘other’ were categorized as ‘other’. The employment status of the responding parent was classified as working fulltime, working part-time, stay-at-home parent, unemployed or other, i.e. studying. Migration background was assessed by country of birth of the responding parent and his/her parents [43]. When either the responding parent or one or both of his/her parents was born outside the Netherlands, this was categorised as a migration background [43].

### Data analysis

Descriptive statistics were used to characterise the participants. Four multivariable linear regression models were used to examine which sociodemographic characteristics were associated with 1) overall perceived social support, 2) support by family members, 3) support by a special person, and 4) support by friends. These four multivariable regression models included all socio-demographic characteristics. Standardised betas (β) and 95%-confidence intervals (CIs) were calculated for each factor. As additional analyses, we explored interactions between socio-demographic characteristics and three potential risk factors identified in previous studies: a low educational level, a migration background, and single-parenthood [26–31]. Interaction terms were separately added to the full multivariable linear regression model for overall support (Additional file 1 Supplementary Table 1). A Bonferroni correction for multiple testing was applied for the interaction analyses (*p* = .05/30 = .002). Stratified analyses were performed for significant interaction effects.

 In addition, multivariable logistic regression models were used to examine associations between sociodemographic characteristics and perceived social support. Odds ratios (ORs) and 95%-confidence intervals (CIs) were calculated for each factor. In all regression models, the source of recruitment was included as a potential confounder.

 Multiple imputation was used to deal with missing values of the socio-demographic characteristics. Missing values varied between 0.2% (*n* = 2) for gender and 6.5% (*n* = 66) for income (Table 1). Five imputed datasets were created for pooled estimates. The linear regression analyses were repeated in the non-imputed dataset, the results were similar (Additional file 1 Supplementary Table 2).

 Data were analysed in Statistical Package for Social Sciences, version 25 for Windows (IBM SPSS Statistics for Windows, IBM Corp). *P*-values below .05 were considered to be statistically significant.

**Table 1:** Characteristics of parents of children aged 0–7 years participating in the CIKEO study (*n* = 1007); by ‘low to moderate’ and ‘high’ levels of perceived social support

	Total	Perceived social support: ‘low to moderate’ (MSPSS score < 5.1)	Perceived social support: ‘high’ (MSPSS score > 5.1)	*P*-value
	*n* = 1007	*n* = 175 (17.4%)	*n* = 832 (82.6%)	
	Mean (SD) n (%)	Mean (SD) n (%)	Mean (SD) n (%)	
*Age of the parent (in years)*	34.1 (SD = 5.1)	35.0 (SD = 5.5)	33.9 (SD = 5.0)	**.017**
*Gender of the parent*				**<.001**
Female	935 (92.9%)70	149 (85.1%)	786 (94.7%)	
Male	(7.0%)	26 (14.9%)	44 (5.3%)	
*Educational level* ^*1*^				**<.001**
High	552 (55.0%)	86 (49.4%)	466 (56.2%)	
Middle	374 (37.3%)	61 (35.1%)	313 (37.8%)	
Low	77 (7.7%)	27 (15.5%)	50 (6.0%)	
*Net monthly household income*				**<.001**
High (>€3200)	615 (65.4%)	85 (50.9%)	530 (68.5%)	
Middle (€2000–€3200)	250 (26.6%)	56 (33.5%)	194 (25.1%)	
Low (<€2000)	76 (8.1%)	26 (15.6%)	50 (6.5%)	
*Employment status*				**<.001**
Part-time job	704 (70,3%)	94 (54.0%)	610 (73.7%)	
Fulltime job	114 (11.4%)	29 (16.7%)	85 (10.3%)	
Stay-at-home parent	132 (13.2%)	29 (16.7%)	103 (12.4%)	
Unemployed	44 (4.4%)	22 (12.6%)	22 (2.7%)	
Other (i.e. studying)	8 (0.8%)	0 (0.0%)	8 (1.0%)	
*Migration background of the parent*				**<.001**
No	860 (85.7%)	135 (77.1%)	725 (87.6%)	
Yes	143 (14.3%)	40 (22.9%)	103 (12.4%)	
*Family situation*				**.006**
Living with a partner	933 (93.2%)	153 (88.4%)	780 (94.2%)	
Living without a partner	68 (6.8%)	20 (11.6%)	48 (5.8%)	
*Number of children*				.959
One child	314 (31.2%)	53 (30.3%)	261 (31.4%)	
Two children	447 (44.4%)	79 (45.1%)	368 (44.2%)	
More than two children	246 (24.4%)	43 (24.6%)	203 (24.4%)	
*Age of the child (in years)*	3.2 (SD = 2.0)	3.6 (SD = 2.2)	3.2 (SD = 1.9)	**.006**
*Gender of the child*				.717
Girl	484 (48.4%)	81 (47.1%)	403 (48.6%)	
Boy	517 (51.6%)	91 (52.9%)	426 (51.4%)	

*P*-values <.05 in bold. *P*-values for continuous variables were based on independent T-tests and *P*-values for categorical variables were based on Chi-squared tests. SD = standard deviation

Missing values: age of the parent *n* = 3; gender parent of the parent *n* = 2; educational level *n* = 4; income *n* = 66; employment status *n* = 5; migration background *n* = 4; family situation *n* = 6; age of the child *n* = 10; gender of the child *n* = 6

^1^Educational level ‘High’: bachelor, master, doctoral or equivalent; ‘Middle’: upper secondary education, post-secondary non-tertiary education, short-cycle tertiary education; ‘Low’: no education, primary education, lower secondary education

### Non-response analysis

Chi-squared tests were used to compare the socio-demographic characteristics of participants who were excluded from the sample for analysis (*n* = 111) with the socio-demographic characteristics of participants included in the sample for analysis (*n* = 1007). No differences were found (*p* > .05) (data not shown).

## Results

### Characteristics of the participants

The characteristics of the population for analysis are presented in Table 1. The mean age of the responding parents was 34.1 years (SD = 5.1); 92.9% were mothers. The majority of the responding parents had a high educational level (55.0%), worked part-time (70.3%), did not have a migration background (85.7%), lived with a partner (93.2%), and had two children (44.4%). The mean age of the child for whom they participated was 3.2 years (SD = 2.0). On average, participants perceived relatively high levels of social support (mean MSPSS score = 5.9; SD = 0.9) [32]. Low levels of perceived social support were more often reported by older parents (p .017), fathers (*p* < .001), parents with a lower educational level (*p* < .001), parents with a lower household income (*p* < .001), parents who were unemployed (*p* < .001), parents with a migration background (*p* < .001), parents living without a partner (*p* = .006), and parents of older children (*p* = .006).

### Associations between socio-demographic characteristics and perceived social support

Table 2 presents the multivariable linear regression models on associations between socio-demographic characteristics and overall perceived social support, support provided by family, support provided by a special person, and support provided by friends. The multivariable regression models include all socio-demographic characteristics. The adjusted R^2^ ranged between 6.9% (support provided by a special person) and 12.2% (overall social support).

**Table 2:** Multivariable linear regression models on associations between socio-demographic characteristics and overall perceived social support and perceived social support by family, a special person and friends among participants of the CIKEO study (n = 1007)

	Overall support	Support provided by family	Support provided by a special person	Support provided by f
	Multivariable model^1^ β (95% CI)	Multivariable model^1^ β (95% CI)	Multivariable model^1^ β (95% CI)	Multivariable model^1^ β (95% CI)
*Age of the parent (in years)*	−0.01 (− 0.08, 0.06)	−0.02 (− 0.09, 0.05)	− 0.06 (− 0.13, 0.01)	0.06 (− 0.01, 0.12)
*Gender of the parent*				
Female	ref.	ref.	ref.	ref.
Male	**− 0.15 (− 0.22, − 0.08)**	**− 0.13 (− 0.20, − 0.06)**	**− 0.10 (− 0.17, − 0.03)**	**− 0.12 (− 0.19, − 0.05)**
*Educational level* ^*2*^				
High	ref.	ref.	ref.	ref.
Middle	0.00 (− 0.06, 0.06)	0.02 (− 0.04, 0.09)	− 0.01 (− 0.07, 0.06)	−0.02 (− 0.08, 0.05)
Low	**−0.12 (− 0.18, − 0.06)**	**−0.11 (− 0.17, − 0.04)**	−0.03 (− 0.09, 0.04)	**−0.15 (− 0.21, − 0.09)**
*Net monthly household income*				
High (>€3200)	ref.	ref.	ref.	ref.
Middle (€2000–€3200)	0.03 (−0.10, 0.04)	−0.01 (− 0.08, 0.06)	−0.02 (− 0.09, 0.05)	−0.04 (− 0.11, 0.03)
Low (<€2000)	**0.10 (− 0.19, − 0.01)**	−0.03 (− 0.12, 0.06)	**−0.10 (− 0.19, − 0.01)**	**−0.12 (− 0.20, − 0.04)**
*Employment status*				
Part-time	ref.	ref.	ref.	ref.
Fulltime	−0.02 (−0.09, 0.05)	0.04 (− 0.03, 0.11)	−0.04 (− 0.11, 0.03)	−0.05 (− 0.12, 0.02)
Stay-at-home parent	−0.06 (− 0.12, 0.01)	0.00 (− 0.07, 0.07)	−0.03 (− 0.09, 0.04)	**−0.11 (− 0.18, − 0.05)**
Unemployed	**−0.14 (− 0.20, − 0.07)**	**−0.10 (− 0.17, − 0.04)**	**−0.08 (− 0.14, − 0.02)**	**−0.14 (− 0.21, − 0.08)**
Other (i.e. studying)	0.05 (− 0.01, 0.11)	0.04 (− 0.02, 0.10)	0.02 (− 0.04, 0.09)	0.05 (− 0.01, 0.12)
*Migration background of the parent*				
No	ref.	ref.	ref.	ref.
Yes	−0.04 (− 0.10, 0.02)	−0.05 (− 0.12, 0.01)	−0.01 (− 0.08, 0.05)	−0.03 (− 0.09, 0.03)
*Family situation*				
Living with a partner	ref.	ref.	ref.	ref.
Living without a partner	0.00 (−0.08, 0.08)	−0.02 (− 0.10, 0.06)	−0.05 (− 0.13, 0.03)	0.07 (0.00, 0.15)
*Number of children in the household*				
One child	ref.	ref.	ref.	ref.
Two children	0.04 (−0.03, 0.12)	0.01 (−0.07, 0.08)	0.07 (− 0.01, 0.14)	0.03 (− 0.04, 0.10)
More than two children	0.04 (− 0.04, 0.11)	0.00 (− 0.07, 0.08)	0.03 (− 0.05, 0.10)	0.06 (− 0.01, 0.13)
*Age of the child (in years)*	−0.07 (− 0.13, 0.00)	**−0.08 (− 0.14, − 0.01)**	−0.07 (− 0.14, 0.00)	−0.01 (− 0.08, 0.06)
*Gender of the child*				
Girl	ref.	ref.	ref.	ref.
Boy	0.01 (−0.05, 0.07)	0.00 (−0.06, 0.06)	0.04 (− 0.02, 0.10)	−0.01 (− 0.06, 0.05)
*Explained variance (adjusted R*^*2*^*)*	12.2%	8.2%	6.9%	11.4%

Table is based on the imputed dataset. Standardized Betas (β) and 95% confidence intervals were derived from the multivariable linear regression models for overall perceived social support and perceived social support provided by family, a special person and friends. *P*-values <.05 in bold. β = Standardized Beta; CI = confidence interval; ref. = reference group.

^1.^The multivariable regression models included the age of the parent, gender of the parent, educational level of the parent, net monthly household income, employment status, number of children in the household, age of the child, gender of the child. All models were additionally adjusted for the source of recruitment

^2.^Educational level ‘High’: bachelor, master, doctoral or equivalent; ‘Middle’: upper secondary education, post-secondary non-tertiary education, short-cycle tertiary education; ‘Low’: no education, primary education, lower secondary education

*Gender of the parent:* compared to mothers, fathers perceived lower levels of overall support (β: -0.15; 95% CI: − 0.22, − 0.08), lower levels of support provided by family (β: -0.13; 95% CI: − 0.20, − 0.06), lower levels of support provided by a special person (β: -0.10; 95% CI: − 0.17, − 0.03), and lower levels of support provided by friends (β: -0.12; 95% CI: − 0.19, − 0.05).

*Educational level:* compared to parents with a high educational level, parents with a low educational level perceived lower levels of overall support (β: -0.12; 95% CI: − 0.18, − 0.06), lower levels of support provided by family (β: -0.11; 95% CI: − 0.17, − 0.04), and lower levels of support provided by friends (β: -0.15; 95% CI: − 0.21, − 0.09).

*Income:* compared to parents with a net household income above €3200/month, parents with a net household income below €2000/month perceived lower levels of overall support (β: -0.10; 95% CI: − 0.19, − 0.01), lower levels of support provided by a special person (β: -0.10; 95% CI: − 0.19, − 0.01), and lower levels of support provided by friends (β: -0.12; 95% CI: − 0.20, − 0.04).

*Employment status:* compared to parents with a part-time job, unemployed parents perceived lower levels of overall support (β: -0.14; 95% CI: − 0.20, − 0.07), lower levels of support provided by family (β: -0.10; 95% CI: − 0.17, − 0.04), lower levels of social support provided by a special person (β: -0.08; 95% CI: − 0.14, − 0.02) and lower levels of support provided by friends (β: -0.14; 95% CI: − 0.21, − 0.08). Stay-at-home parents perceived lower levels of support provided by friends (β: 0.11; 95% CI: − 0.18, − 0.05) compared to parents with a part-time job.

*Living situation:* Parents living with and without a partner perceived similar levels of overall support, and similar levels of support provided by family a special person. Compared to parents living with a partner, parents living without a partner perceived higher levels of support provided by friends (β: 0.07; 95% CI: 0.00, 0.15).

*Age of the child:* Parents of older children perceived lower levels of overall social support (β: -0.07; 95% CI: − 0.13, 0.00), lower levels of support provided by family (β: -0.08; 95% CI: − 0.14, − 0.01), and lower levels of support provided by a special person (β: -0.07; 95% CI: − 0.14, 0.00).

Age of the parent, the number of children in the household, and the gender of the child were not associated with parents’ perceived social support (*p* > .05) (Table 2).

### Additional analyses: interaction effects

Additional file 1 Supplementary Table 1 presents the results of the interaction analyses. Significant interaction effects were found between living situation and unemployment (*p* < .001) and between the parent's migration background and educational level (*p* = .002).

*Living situation and unemployment:* Among parents living with a partner (*n* = 933) unemployment was associated with lower levels of perceived social support, while among parents living without a partner (*n* = 68) unemployment was not associated with perceived social support. However, this finding was based on a small number of participants who were living without a partner and unemployed (*n* = 12). Therefore, the interaction effect between living situation and unemployment has not been examined further in this study.

*Migration background and educational level:* Table 3 presents the fully adjusted multivariable linear regression models on the association between educational level and perceived social support, stratified by migration background. Among parents with a migration background (*n* = 144), having a low educational level was associated with lower levels of overall support (β: 0.34; 95% CI: − 0.52, − 0.15), lower levels of support provided by family (β: -0.27; 95% CI: − 0.48, − 0.07), lower levels of support provided by a special person (β: -0.26; 95% CI: − 0.44, − 0.07), and lower levels of support provided by friends (β: -0.28; 95% CI: − 0.46, − 0.10). Among parents without a migration background (*n* = 863), having a low educational level was associated with lower levels of overall support (β: -0.07; 95% CI: − 0.14, 0.00), and lower levels of support provided by friends (β: -0.12; 95% CI: − 0.19, − 0.05).

**Table 3:** Multivariable linear regression models on the associations between educational level and perceived social support among 1007 participants of the CIKEO study; stratified by migration background of the responding parent

	Overall support^1^		Support provided by family^1^		Support provided by a special person^1^		Support provided by friends^1^	
	Migration background		Migration background		Migration background		Migration background	
	Yes (*n* = 144)	No (*n* = 863)	Yes (*n* = 144)	No (*n* = 863)	Yes (*n* = 144)	No (*n* = 863)	Yes (*n* = 144)	No (*n* = 863)
	β (95% CI)	β (95% CI)	β (95% CI)	β (95% CI)	β (95% CI)	β (95% CI)	β (95% CI)	β (95% CI)
Educational level of the responding parent^2^								
High	ref.	ref.	ref.	ref.	ref.	ref.	ref.	ref.
Middle	−0.03 (−0.24, 0.19)	−0.03 (−0.07, 0.06)	0.08 (−0.16, 0.32)	0.00 (− 0.06, 0.07)	0.01 (− 0.20, 0.21)	−0.01 (− 0.08, 0.06)	−0.16 (− 0.37, 0.05)	−0.00 (− 0.07, 0.07)
Low	**− 0.34 (− 0.52, − 0.15)**	**−0.07 (− 0.14, 0.00)**	**−0.27 (− 0.48, − 0.07)**	−0.07 (− 0.13, 0.00)	**−0.26 (− 0.44, − 0.07)**	0.02 (− 0.06, 0.09)	**−0.28 (− 0.46, − 0.10)**	**−0.12 (− 0.19, − 0.05)**

Table is based on the imputed dataset. β and 95% confidence intervals were derived from the multivariable linear regression models for overall perceived social support. *P*-values <.05 in bold. β = Standardized Beta; CI = confidence interval; ref. = reference group

^1.^ The multivariable models were adjusted for age of the parent, gender of the parent, net monthly household income, employment status, number of children in the household, age of the child, gender of the child and the source of recruitment

^2. ^Educational level ‘High’: bachelor, master, doctoral or equivalent; ‘Middle’: upper secondary education, post-secondary non-tertiary education, short-cycle tertiary education; ‘Low’: no education, primary education, lower secondary education

### Additional analyses: logistic regression

Additional file 1 Supplementary Table 2 presents the results of the logistic regression analyses of the associations between socio-demographic characteristics and perceived social support (‘low to moderate’/ ‘high’). The results were similar to the results of the linear regression analyses. Fathers (OR: 2.75; 95% CI: 1.41, 5.37), parents with a low educational level (OR: 2.07; 95% CI: 1.14, 3.77), and unemployed parents (OR: 3.95; 95% CI: 1.93, 8.10), had higher odds of perceiving ‘low to moderate’ levels of social support.

## Discussion

This study examined which socio-demographic characteristics are associated with perceived social support among parents of children aged 0–7 years. On average, participants perceived relatively high levels of social support (M = 5.9; SD = 0.9) [40]. In other studies among general populations of parents, mean MSPSS scores were 5.2 (SD = 1.3) [44] and 6.0 (SD = 1.2) [45]. We found that fathers, parents with a low educational level, parents with a low income, unemployed parents, and parents of older children perceived lower levels of social support. Interaction analyses showed that parents with both a migration background and a low educational level were particularly susceptible to perceiving lower levels of social support. Below, these findings are discussed in more detail.

Fathers perceived lower levels of social support. This finding is in line with the results of several previous studies among adults in which women reported greater access to social support [46–48]. Studies on social support among parents have mainly been conducted among mothers [26, 27, 29, 30, 49]. Although we included fathers, only 7.0% (*n* = 70) of the participants were fathers, and this sample may not be representative for the total population. To gain a better understanding of gender differences in perceived social support among parents, we recommend to pay special attention to the inclusion of fathers in future study samples. For example, by inviting both parents to participate.

Parents with a low educational level and parents with a low income perceived lower levels of social support. This finding is in line with the results of previous studies [26, 27]. Having a low socioeconomic position may reduce access to social support for several reasons [26, 27, 49, 50]. First, parents with a low socioeconomic position may be embedded in resource-poor networks, comprised of people with a similar socioeconomic status, with fewer opportunities to provide support [26, 27, 49, 50]. Secondly, parents with a low socioeconomic position may be less able to reciprocate support, which, in turn, may negatively influence their chances of receiving support [51]. Further explanations include possible financial barriers to participation in social activities, poverty-related stigmatization and shame related to withdrawal from social activities [52].

Unemployed parents perceived lower levels of social support, which is in line with previous findings [27, 30]. Parents without a job may have fewer opportunities to expand their social network [51]. At the same time, access to social support may influence parents’ opportunities to work by instrumental support such as providing transportation or child care assistance during irregular work hours [30].

Living without a partner was not associated with overall social support, which contradicts previous findings [26, 30]. Our findings may differ because we used a different measure to assess perceived social support, including different dimensions of social support and different sources of support. We found that parents living without a partner perceived higher levels of support provided by friends. This is in line with findings from a previous study which indicated that single parents may receive additional support from their friends and family [53].

Parents of older children (age range 0–7 years) perceived lower levels of social support. Several previous studies did not examine this association [26–31], but Harknett and Hartnett [26] found that older parents perceived lower levels of social support. Future studies may examine whether parents’ need for social support and access to social support may differ by the age stage of their child.

Interaction analyses showed that parents with both a migration background and a low educational level were particularly susceptible to perceiving lower levels of social support. Previous studies suggest that transnational migration may have a negative influence on perceived social support by a dislocation of social networks, language barriers, cultural differences, and feelings of marginality [26, 31]. Based on our findings, we hypothesize that obtaining a high educational level might enhance social integration and active participation in society [54] and might thereby reduce the negative impact of migration on perceived social support.

There is a need to gain a better understanding of why the abovementioned subgroups of parents are at risk of perceiving a lack of social support. Perceiving a lack of social support may, for example, be related to a higher need for support and/ or to a reduced availability of social support [26]. Knowing why parents perceive a lack of social support may clarify what type of interventions are needed, for example interventions that increase the availability of social support or interventions that educate parents on how to mobilize available social support [55]. Future studies should examine this. Qualitative research may be helpful to gain more in-depth insights into why these groups of parents are at risk of perceiving lower levels of social support [56].

In additional multivariable linear regression analyses, we explored the potential role of the health of the child by adding a variable on the general health status of the child to the models. The regression models are presented in Additional file 1 Supplementary Table 4. Parents of children with a poorer general health status perceived lower levels of overall social support, and lower levels of social support provided by family, a special person and friends. The associations between socio-demographic characteristics and perceived social support were similar after adjusting for the general health status of the child. Associations between social support and health are likely to be bi-directional. Health problems may increase the need for social support, but social support has also been associated with positive outcomes for health [1–7]. We advise to further examine the role of child and parental health in future research.

### Methodological considerations

This study adds to the literature by its relatively large sample size and the use of a validated measure which included the appraisal and emotional dimension of social support and distinguished between different sources of support. Several limitations of our study should be taken into account. First, a comparison of the participants’ socio-demographic characteristics with national open data [57] showed that parents with a low educational level, parents with lower income levels, parents with a migration background, and parents living without a partner were relatively underrepresented in the sample. Although the statistical power to detect associations may have been reduced by this underrepresentation, we have no rationale to expect that the directions of the associations have been affected. Future studies may expand upon our findings by using large and diverse samples of mother and fathers. Second, causality could not be inferred due to the cross-sectional nature of this study. Several associations, including the association between a parents’ socio-economic position and perceived social support may be bi-directional. A parent’ s socio-economic position, for example, may be related to lower perceived social support, but the lack of a resourceful network may also reduce opportunities to achieve a higher socioeconomic position, as social networks may offer access to training and job opportunities [26, 49, 51]. Future studies may expand upon our findings by examining the directions of these associations. 

As a methodological consideration, we want to point out that surveys completed by two parents together (*n* = 34) were excluded as we were interested in their individual experience of social support. However, some parents may have difficulties completing a questionnaire and may require help from their partner. As a sensitivity analysis, we included these parents in the sample and repeated the regression analyses (data not shown). All associations were similar, which indicates that excluding these parents did not influence the results of this study.

### Implications for practice and policy

Social support is important for families’ health, wellbeing and empowerment to deal with parenting issues. This study identified groups of parents at risk of perceiving low levels of support. To ensure that parents feel supported, policy makers, health and social care professionals should pay special attention to groups of parents who perceive lower levels of social support. In conversations with parents, professionals may actively listen to parents’ experiences with social support to gain a better understanding of their needs and strengths [58]. Various intervention strategies may be used to strengthen social support [33, 59, 60]. First, professionals may approach members of the existing social network to mobilise informal support and community resources [59–61]. Second, communication and social skills training may be used to improve a parent’s ability to ask for support and to receive support [33, 60]. Third, professionals may facilitate parent groups [20, 59]. Dialogues about parenting may stimulate parents to reflect on their parenting style, and to exchange support and advice [20]. When organised in local communities, parents may continue to meet each other, and form self-sustaining social networks [20, 59].

## Conclusion

Perceived social support among parents of children aged 0–7 years is unevenly distributed. Fathers, parents with a low educational level, parents with a low household income, unemployed parents and parents of older children are at increased risk of perceiving lower levels of social support. Parents with both a migration background and a low educational level may be particularly susceptible of perceiving lower levels of social support. Longitudinal research in diverse populations is needed to confirm these findings. Qualitative research may provide insight into why these groups of parents are at increased risk of perceiving a lack of social support. In the meantime, policy makers, health and social care professionals should be aware of the increased susceptibility to lower levels of perceived social support among the abovementioned subgroups of parents. We recommend to develop, implement and evaluate intervention strategies that strengthen perceived social support among parents in order to improve their families’ health and wellbeing and to empower them to deal with parenting issues.

## Supplementary Information

**Additional file 1.** Supplementary tables.

## Data Availability

The dataset analysed during the current study is not publicly available due to ethical restrictions with regard to the privacy of research participants but are available from the corresponding author on reasonable request.
