# Supplementary material for: Socio-demographic characteristics associated with perceived social support among parents of children aged 0–7 years: the CIKEO study
Source: BMC Public Health. 2022 Dec 27;22:2441. doi: 10.1186/s12889-022-14830-1 (PMC9795715; doi:10.1186/s12889-022-14830-1)
Supplement: Supplementary file 1 — Additional file 1. Supplementary tables. [file 12889_2022_14830_MOESM1_ESM.docx]

# Supplementary file Socio-demographic characteristics associated with perceived social support among parents of children aged 0-7 years: the CIKEO study

**Supplementary Table 1** Results of the interaction analyses for socio-demographic characteristics associated with overall perceived social support among participants of the CIKEO study (n=1007).

| **Interaction term** | **P-value of the Standardized Beta of the interaction term in the full multivariable linear regression model for overall perceived social support (MSPSS)^1^** | **Adjusted R^2^ of the multivariable linear regression model including the interaction term^2^** |
| --- | --- | --- |
| Living without a partner*age of the parent | .229 | 12.2% |
| Living without a partner*gender of the parent | .024 | 12.6% |
| Living without a partner*low educational level | .639 | 12.1% |
| Living without a partner*low income | .250 | 12.2% |
| Living without a partner*working fulltime | .196 | 12.2% |
| Living without a partner*stay-at-home parent | .034 | 12.6% |
| Living without a partner*unemployed | **<.001** | 13.1% |
| Living without a partner*migration background | .623 | 12.1% |
| Living without a partner*number of children | .549 | 12.1% |
| Living without a partner*age of the child | .143 | 12.3% |
| Living without a partner*gender of the child | .944 | 12.1% |
| Low educational level*age of the parent | .067 | 12.4% |
| Low educational level*gender of the parent | .153 | 12.6% |
| Low educational level*low income | .140 | 12.3% |
| Low educational level*working fulltime | .947 | 12.1% |
| Low educational level*stay-at-home parent | .159 | 12.3% |
| Low educational level*unemployed | .010 | 12.7% |
| Low educational level*migration background | **.002** | 12.9% |
| Low educational level*number of children | .737 | 12.1% |
| Low educational level*age of the child | .561 | 12.1% |
| Low educational level*gender of the child | .519 | 12.1% |
| Migration background*age of the parent | .130 | 12.3% |
| Migration background*gender of the parent | .049 | 12.4% |
| Migration background*low income | .410 | 12.2% |
| Migration background*fulltime job | .073 | 12.4% |
| Migration background*stay-at-home parent | .077 | 12.4% |
| Migration background*unemployed | .911 | 12.1% |
| Migration background*number of children | .691 | 12.1% |
| Migration background*age of the child | .654 | 12.1% |
| Migration background*gender of the child | .345 | 12.1% |

Table is based on the imputed dataset. A Bonferroni correction for multiple testing was applied for the interaction analyses (p=.05/30=.002).

^1^ P-values of the standardized Betas were derived by separately adding the interaction terms to the (Table 2).
^2^ For comparison: the adjusted R^2^ of the model multivariable linear regression model for overall perceived social support without the interaction term was 12.2%.

**Supplementary Table 2** Sensitivity analysis using the non-imputed dataset: Multivariable linear regression models on associations between socio-demographic characteristics and overall perceived social support and perceived social support by family, a special person and friends among participants of the CIKEO study (n=1007).

|  | **Overall support** | **Support provided by family** | **Support provided by a special person** | **Support provided by friends** |
| --- | --- | --- | --- | --- |
|  | Multivariable model^1^ β (95% CI) | Multivariable model^1^  β (95% CI) | Multivariable model^1^  β (95% CI) | Multivariable model^1^ β (95% CI) |
| *Age of the parent (in years)* | 0.00 (-0.07, 0.07) | -0.02 (-0.09, 0.06) | -0.07 (-0.14, 0.01) | **0.07 (0.00, 0.15)** |
| *Gender of the parent*  Female   Male | ref. **-0.16 (-0.23, -0.09)** | ref. -**0.13 (-0.21, -0.06)** | ref. **-0.11 (-0.18, -0.04)** | ref. **-0.14 (-0.21, -.07)** |
| *Educational level ^2^*  High  Middle  Low | ref. -0.01 (-0.07, 0.06)  **-0.11 (-0.18, -0.04)** | ref. 0.02 (-0.04, 0.09)  **-0.11 (-0.18, -0.04)** | ref. -0.02 (-0.09, 0.05)  -0.04 (-0.11, 0.03) | ref. -0.02 (-0.08, 0.05)  **-0.12 (-0.19, -0.05)** |
| *Net monthly household income*   High (>€3200)  Middle (€2000-€3200)  Low (<€2000) | ref. -0.04 (-0.11, 0.03)  **-0.10 (-0.19, -0.01)** | ref. -0.01 (-0.09, 0.06)  -0.03 (-0.12, 0.06) | ref.  -0.02 (-0.09, 0.05)  -0.07 (-0.16, 0.02) | ref. -0.05 (-0.12, 0.02)  **-0.14 (-0.23, -0.05)** |
| *Employment status*  Part-time  Fulltime  Stay-at-home parent  Unemployed  Other (i.e. studying) | ref. -0.01 (-0.08, 0.06)  -0.06 (-0.13, 0.01)  **-0.13 (-0.20, -0.07)**  0.06 (-0.01, 0.12) | ref. 0.03 (-0.04, 0.11)  -0.01 (-0.08, 0.06)  **-0.11 (-0.18, -0.04)**  0.05 (-0.02, 0.11) | ref. -0.03 (-0.10, 0.04)  -0.03 (-0.09, 0.04)  **-0.10 (-0.16, -0.03)**  0.04 (-0.03, 0.10) | ref. -0.04 (-0.11, 0.03)  **-0.11 (-0.17, -0.04)**  **-0.11 (-0.18, -0.04)**  0.05 (-0.01, 0.12) |
| *Migration background of the parent*  No  Yes | ref. -0.05 (-0.12, 0.01) | ref. -0.06 (-0.12, 0.01) | ref. -0.02 (-0.08, 0.05) | ref. -0.05 (-0.11, 0.02) |
| *Family situation*  Living with a partner  Living without a partner | ref. 0.00 (-0.08, 0.08) | ref. -0.03 (-0.11, 0.06) | ref. -0.06 (-0.14, 0.02) | ref. **0.09 (0.01, 0.17)** |
| *Number of children in the household*   One child  Two children  More than two children | ref. 0.04 (-0.03, 0.12)  0.05 (-0.03, 0.13) | ref. 0.00 (-0.07, 0.08)  0.01 (-0.07, 0.10) | ref. 0.07 (-0.01, 0.14)  0.03 (-0.05, 0.11) | ref. 0.04 (-0.04, 0.11)  **0.08 (0.00, 0.16)** |
| *Age of the child (in years)* | **-0.08 (-0.15, -0.01)** | **-0.09 (-0.16, -0.02)** | **-0.08 (-0.15, 0.00)** | -0.03 (-0.10, 0.04) |
| *Gender of the child*  Girl  Boy | ref. 0.01 (-0.06, 0.07) | ref. -0.01 (-0.07, 0.06) | ref. 0.02 (-0.04, 0.08) | ref. 0.00 (-0.06, 0.06) |
| *Explained variance (adjusted R^2^)* | 11.4% | 8.5% | 6.7% | 9.8% |

Table is based on the non-imputed dataset. Standardized Betas (β) and 95% confidence intervals were derived from the multivariable linear regression models for overall perceived social support and perceived social support provided by family, a special person and friends. P-values <.05 in bold. β=Standardized Beta; CI=confidence interval; ref.=reference group.
^1.^ The multivariable regression models included the age of the parent, gender of the parent, educational level of the parent, net monthly household income, employment status, number of children in the household, age of the child, gender of the child. All models were additionally adjusted for the source of recruitment.

^2.^ Educational level ‘High’: bachelor, master, doctoral or equivalent; ‘Middle’: upper secondary education, post-secondary non-tertiary education, short-cycle tertiary education; ‘Low’: no education, primary education, lower secondary education.

**Supplementary Table 3** Multivariable logistic regression models on associations between socio-demographic characteristics and overall perceived social support and perceived social support by family, a special person and friends among participants of the CIKEO study (n=1007).

|  | **‘Low to moderate’ overall perceived social support (MSPSS)**  ‘yes’ n=175 (17.4%) | **‘Low to moderate’ support provided by family**  ‘yes’ n=214 (21.3%) | **‘Low to moderate’ support provided by a special person**  ‘yes’ n=142 (14.1%) | **‘Low to moderate’ support provided by friends**  ‘yes’ n=232 (23.0%) |
| --- | --- | --- | --- | --- |
|  | Multivariable model^1^ OR (95% CI) | Multivariable model^1^  OR (95% CI) | Multivariable model^1^  OR (95% CI) | Multivariable model^1^  OR (95% CI) |
| *Age (in years)* | 1.01 (0.97, 1.04) | 1.00 (0.96, 1.03) | 1.03 (0.99. 1.07) | 0.97 (0.93. 1.00) |
| *Gender*  Female   Male | ref.  **2.75 (1.41, 5.37)** | ref. **2.29 (1.20, 4.37)** | ref.  **1.96 (1.00, 3.83)** | ref. **2.07 (1.09, 3.92)** |
| *Educational level^2^*  High  Middle  Low | ref.  0.97 (0.65, 1.46)  **2.07 (1.14, 3.77)** | ref. 0.84 (0.58, 1.20)  1.43 (0.80, 2.56) | ref.  0.95 (0.62, 1.45)  1.69 (0.88, 3.23) | ref. 1.08 (0.76, 1.55)  **2.96 (1.71, 5.13)** |
| *Net monthly household income*   High (>€3200)  Middle (€2000-€3200)  Low (<€2000) | ref. 1.41 (0.92, 2.17)  2.11 (0.92, 4.85) | ref. 1.20 (0.80, 1.79)  1.27 (0.54, 2.98) | ref.  1.03 (0.64, 1.67)  2.06 (0.91, 4.65) | ref. **1.63 (1.08, 2.46)**  1.88 (0.90, 3.95) |
| *Employment status*  Part-time  Fulltime  Stay-at-home parent  Unemployed  Other (i.e. studying) | ref. 1.31 (0.73, 2.37)  1.36 (0.81, 2.29)  **3.95 (1.93, 8.10) -** | ref.  0.92 (0.53, 1.62)  0.77 (0.45, 1.30)  **2.56 (1.25, 5.23)** - | ref. 1.56 (0.86, 2.83)  1.27 (0.71, 2.25)  1.57 (0.70, 3.51)  - | ref. 1.67 (0.99, 2.84)  **1.65 (1.04, 2.63)**  **2.43 (1.20, 4.93)** - |
| *Migration background*  No  Yes | ref. 1.43 (0.89, 2.29) | ref. 1.09 (0.70, 1.72) | ref. 1.32 (0.80, 2.17) | ref. 1.23 (0.79, 1.92) |
| *Family composition*  Living with a partner  Living without a partner | ref.  0.75 (0.34, 1.68) | ref. 0.78 (0.35, 1.73) | ref.  1.21 (0.55, 2.66) | ref. 0.75 (0.36, 1.59) |
| *Number of children in the household*  One child  Two children  More than two children | ref. 1.00 (0.65, 1.54)  0.95 (0.57, 1.59) | ref. 1.04 (0.71, 1,55)  1.16 (0.73, 1,84) | ref.  0.81 (0.51, 1.27)  0.98 (0.58, 1.67) | ref. 0.92 (0.63, 1.34)  0.80 (0.51, 1.27) |
| *Age of the child (in years)* | 1.09 (0.99, 1.20) | 1.07 (0.98, 1,17) | 1.02 (0.92, 1.13) | 1.01 (0.93, 1.11) |
| *Gender of the child*  Girl  Boy | ref. 1.01 (0.71, 1.44) | ref. 0.93 (0.68, 1.27) | ref. 0.86 (0.60, 1.25) | ref. 1.11 (0.81, 1.52) |

Table is based on the imputed dataset. OR and 95% confidence intervals were derived from the multivariable logistic regression models for overall perceived social support and perceived social support provided by family, a special person and friends. P-values <.05 in bold. OR=Odds Ratio; CI=confidence interval; ref.=reference group.
All models were adjusted for the source of recruitment.
^1.^ The multivariable regression models included the age of the parent, gender of the parent, educational level of the parent, net monthly household income, employment status, number of children in the household, age of the child, gender of the child. All models were additionally adjusted for the source of recruitment.
^2.^ Educational level ‘High’: bachelor, master, doctoral or equivalent; ‘Middle’: upper secondary education, post-secondary non-tertiary education, short-cycle tertiary education; ‘Low’: no education, primary education, lower secondary education.

**Supplementary Table 4** Additional analyses: Multivariable linear regression models on associations between socio-demographic characteristics and overall perceived social support and perceived social support by family, a special person and friends among participants of the CIKEO study (n=1007); adjusted for the general health status of the child.

|  | **Overall support** | **Support provided by family** | **Support provided by a special person** | **Support provided by friends** |
| --- | --- | --- | --- | --- |
|  | Multivariable model^1^ β (95% CI) | Multivariable model^1^  β (95% CI) | Multivariable model^1^  β (95% CI) | Multivariable model^1^ β (95% CI) |
| *Age of the parent (in years)* | -0.01 (-0.08, 0.06) | -0.02 (-0.09, 0.06) | -0.07 (-0.14, 0.01) | 0.06 (-0.01, 0.13) |
| *Gender of the parent*  Female   Male | ref. **-0.16 (-0.22, -0.09)** | ref. **-0.14 (-0.21, -0.07)** | ref. **-0.11 (-0.18, -0.04)** | ref. **-0.13 (-0.20, -0.06)** |
| *Educational level ^2^*  High  Middle  Low | ref. 0.00 (-0.06, 0.06)  **-0.12 (-0.18, -0.05)** | ref. 0.02 (-0.04, 0.09)  **-0.10 (-0.17, -0.04)** | ref. -0.00 (-0.07, 0.06)  -0.03 (-0.10, 0.04) | ref. -0.02 (-0.08, 0.05)  **-0.14 (-0.21, -0.08)** |
| *Net monthly household income*   High (>€3200)  Middle (€2000-€3200)  Low (<€2000) | ref. -0.03 (-0.09, 0.04)  **-0.09 (-0.18, 0.00)** | ref.  -0.01 (-0.08, 0.06)  -0.02 (-0.11, 0.08) | ref. -0.02 (-0.09, 0.05)  **-0.09 (-0.18, 0.00)** | ref. -0.04 (-0.11, 0.03)  **-0.11 (-0.19, -0.02)** |
| *Employment status*  Part-time  Fulltime  Stay-at-home parent  Unemployed  Other (i.e. studying) | ref. -0.02 (-0.09, 0.05)  -0.06 (-0.13, 0.00)  **-0.14 (-0.20, -0.07)**  0.05 (-0.01, 0.11) | ref. 0.04 (-0.03, 0.11)  -0.01 (-0.07, 0.06)  **-0.11 (-0.17, -0.04)**  0.04 (-0.02, 0.10) | ref. -0.04 (-0.11, 0.03)  -0.03 (-0.09, 0.04)  **-0.09 (-0.15, -0.02)**  0.03 (-0.04, 0.09) | ref. -0.05 (-0.12, 0.02)  **-0.11 (-0.18, -0.05)**  **-0.13 (-0.20, -0.07)**  0.06 (0.00, 0.12) |
| *Migration background of the parent*  No  Yes | ref. -0.04 (-0.10, 0.02) | ref. -0.05 (-0.11, 0.01) | ref. -0.01 (-0.07, 0.05) | ref. -0.03 (-0.09, 0.03) |
| *Family situation*  Living with a partner  Living without a partner | ref. -0.01 (-0.08, 0.07) | ref. -0.03 (-0.11, 0.05) | ref. -0.05 (-0.13, 0.03) | ref. 0.06 (-0.01, 0.14) |
| *Number of children in the household*   One child  Two children  More than two children | ref. 0.06 (-0.02, 0.13)  0.05 (-0.03, 0.12) | ref. 0.02 (-0.05, 0.09)  0.01 (-0.06, 0.09) | ref. **0.08 (0.00, 0.15)**  0.04 (-0.04, 0.11) | ref. 0.05 (-0.03, 0.12)  0.07 (-0.01, 0.14) |
| *Age of the child (in years)* | -0.06 (-0.12, 0.01) | -0.07 (-0.14, .00) | -0.06 (-0.13, 0.01) | -0.01 (-0.08, 0.06) |
| *Gender of the child*  Girl  Boy | ref. 0.01 (-0.05, 0.07) | ref. 0.00 (-0.06, 0.06) | ref. 0.04 (-0.03, 0.10) | ref. 0.00 (-0.06, 0.06) |
| *General health status of the child*  *(better health)*^3^ | **0.12 (0.06, 0.18)** | **0.12 (0.06, 0.18)** | **0.08 (0.02, 0.14)** | **0.09 (0.04, 0.15)** |
| *Explained variance (adjusted R^2^)* | 13.2% | 9.3% | 7.6% | 11.5% |

Table is based on the non-imputed dataset. Standardized Betas (β) and 95% confidence intervals were derived from the multivariable linear regression models for overall perceived social support and perceived social support provided by family, a special person and friends. P-values <.05 in bold. β=Standardized Beta; CI=confidence interval; ref.=reference group.
^1.^ The multivariable regression models included the age of the parent, gender of the parent, educational level of the parent, net monthly household income, employment status, number of children in the household, age of the child, gender of the child. All models were additionally adjusted for the source of recruitment.
^2.^ Educational level ‘High’: bachelor, master, doctoral or equivalent; ‘Middle’: upper secondary education, post-secondary non-tertiary education, short-cycle tertiary education; ‘Low’: no education, primary education, lower secondary education.
^3^ General health of the child was measured with the first item of the Child Health Questionnaire (1); Scores range between 0-100: 0 indicates poor health; 100 indicates excellent health. Missing values general health status of the child n=12.

1. Raat H, Botterweck AM, Landgraf JM, Hoogeveen WC, Essink-Bot M-L. Reliability and validity of the short form of the child health questionnaire for parents (CHQ-PF28) in large random school based and general population samples. Journal of Epidemiology & Community Health. 2005;59(1):75-82.
